# Effects of Stocking Density on the Growth Performance, Physiological Parameters, Redox Status and Lipid Metabolism of *Micropterus salmoides* in Integrated Rice–Fish Farming Systems

**DOI:** 10.3390/antiox11071215

**Published:** 2022-06-21

**Authors:** Rui Jia, Long Wang, Yiran Hou, Wenrong Feng, Bing Li, Jian Zhu

**Affiliations:** 1 Key Laboratory of Integrated Rice–Fish Farming Ecology, Ministry of Agriculture and Rural Affairs, Freshwater Fisheries Research Center, Chinese Academy of Fishery Sciences, Wuxi 214081, China; jiar@ffrc.cn (R.J.); houyr@ffrc.cn (Y.H.); fengwenrong@ffrc.cn (W.F.); 2 International Joint Research Laboratory for Fish Immunopharmacology, Freshwater Fisheries Research Center, Chinese Academy of Fishery Sciences, Wuxi 214081, China; 3 Wuxi Fisheries College, Nanjing Agricultural University, Wuxi 214081, China; 2020813069@stu.njau.edu.cn

**Keywords:** oxidative stress, physiological stress response, transcriptome, PPAR signaling pathway

## Abstract

Stocking density has been identified as one of the main factors affecting fish growth, welfare and behavior. However, few studies have focused on the effects of stocking density on fish health in integrated rice–fish farming systems. Thus, the aim of this study was to evaluate the effects of different stocking densities on the growth performance, physiological parameters, redox status and lipid metabolism of *Micropterus salmoides* in an integrated rice–fish farming system. The fish were reared at three densities: low density (LD, 40 g/m^3^), medium density (MD, 80 g/m^3^) and high density (HD, 120 g/m^3^) for 90 days. At the end of the experiment, fish reared in the MD and HD groups showed lower growth performance than those from the LD group. The HD treatment significantly altered the physiological parameters, including glucose and lactate. Meanwhile, the HD treatment induced oxidative stress and lipid peroxidation after 90 days of farming. Furthermore, transcriptomic analysis revealed that HD treatment led to abnormal lipid metabolism. Interestingly, we found the suppression of three key pathways related to lipid metabolism, including the PPAR, insulin and adipocytokine signaling pathways, in the HD group. Overall, our data indicated that the HD treatment inhibited growth and caused physiological responses, oxidative stress and abnormal hepatic lipid metabolism in *M. salmoides* in an integrated rice–fish farming system.

## 1. Introduction

Integrated rice–fish farming is an environmentally friendly agriculture model developed from traditional fish culture in paddies due to its environmental sustainability and food productivity [1]. In China, it is expanding rapidly and has become one of the major food production approaches, having an area of 1,793,409 ha and aquatic production of 3254 thousand tons in 2020 [2]. Integrated rice–fish farming is also considered as an ecosystem-based approach which can achieve the complementary utilization of wetland, arable land, freshwater and aquatic living resources [3]. In the system, the rice field provides potential food for fish (crab or shrimp) such as benthos, phytoplankton and zooplankton, while fish (crab or shrimp) feces can be absorbed into the soil as fertilizer [4]. Moreover, the biomass of weeds and planthoppers are effectively reduced in rice fields because of the consumption of aquatic plants, pests and insects by the farmed animals [5]. Rice–fish co-culture also alters the diversity and biomass of phytoplankton and zooplankton in the system. Phytoplankton communities and protozoan numbers are increased in rice–fish systems due to the higher availability of nutrients through fish defecation and perturbation [6]; zooplankton and benthic invertebrates are decreased due to their utilization as food by fish in the system [7]. Furthermore, ecosystem service analyses have indicated that the value of rice–fish farming is 38% greater than that of rice monoculture [8]. Thus integrated rice–fish farming may be a sustainable alternative to rice monoculture by increasing food production, improving economic benefit and reducing environmental impact [3,9]. However, its broad practice may be limited by some existing problem, such as water management, technical and institutional constraints, adequate farming density and so on [10,11].

Stocking density has been identified as one of the main factors affecting fish growth, welfare and behavior [12]. In commercial aquaculture, the optimum stocking density of fish is set according to their physiological and biological characteristics, and the economic benefit of the farm. Numerous studies have reported that higher stocking densities inhibited the growth performance of many farmed fish, such as *Scophthalmus maximus*, *Oreochromis niloticus* and *Salmo salar* [13,14,15]. High stocking density is also considered to be a stress factor which may cause a negative change in physiological and biochemical parameters, reduce immunological function and increase the risk of disease outbreaks in fish [16,17,18]. Furthermore, a high stocking density induced oxidative stress and oxidative damage in fish [19,20]. In integrated rice–fish farming systems, the effects of stoking density on productivity, soil fertility and the environment have primarily been assessed [21]; for example, Vongvichith et al. (2018) reported that stocking density was a primary factor of fish productivity, and productivity was reduced at a higher stocking density [22]. Li et al. (2007) found that the specific growth rate (SGR) and survival rate (SR) of *Eriocheir sinensis* were significantly lower at the higher stocking density in a rice–crab co-culture system [23]. However, few studies have currently been performed on the physiological, biochemical and molecular variations of aquatic animals under different stocking densities in integrated rice–fish farming systems.

The largemouth bass (*Micropterus salmoides*) has been extensively cultured in China since its introduction in the 1980s and has become an important freshwater farmed species with a production of 619,519 tons in 2020 [24]. Pond culture is the main production method for largemouth bass [25]. In addition, it is raised in recirculating aquaculture systems and in-pond raceway systems [26,27]. The amenable stocking density of largemouth bass is different in different farming models, such as at least 0.15 kg/m^3^ in ponds (12 months of farming), 6.85 kg/m^3^ in land-based recirculating aquaculture systems (90 days of farming) and 4.2 kg/m^3^ in in-pond raceway systems (150 days of farming) [28,29,30]. The adverse effects of a high stocking density on growth performance, biochemical parameters and immune response have been frequently reported in largemouth bass under various farming models [27,28]. In recent years, rice–largemouth bass co-culture, as a new farming model, has been attempted in China and has obtained a positive economic benefit [31,32]. However, the optimum stocking density of largemouth bass is still unclear, and the effects of high stocking density on growth performance, physiological response and molecular function have not been reported in this system.

In the current investigation, we aimed to evaluate the effects of stocking density on the growth performance, physiological parameters, redox status and hepatic lipid metabolism of largemouth bass in an integrated rice–fish farming system. For this purpose, the fish were raised in an integrated rice–fish farming system at three densities for 90 days. We then measured the growth performance, physiological blood parameters, antioxidant status and hepatic transcriptome. The data will provide a reference for largemouth bass farming and optimization of the rice–fish farming model.

## 2. Materials and Methods

### 2.1. Rice–Fish Farming System, Experimental Design and Sampling

The study was carried out at the experimental base of the Freshwater Fisheries Research Center (32°08′ N, 120°19′ E) belonging to a subtropical monsoon climate with an annual rainfall of 1078 mm and a mean temperature of 16–20 °C. The rice–fish farming system includes a rice planting area (1624 m^2^) and a fish stocking area (0.8 m in depth, 176 m^2^) (Figure S1). In the system, rice seedlings (Nangeng 5055) were transplanted in the middle of June 2021, and the rice was finally harvested in early November; the fish fingerlings were stocked on 30 July and harvested on 30 October. The management of the paddy field was performed according to the local conventional agricultural practices [33,34]. In addition to rice and fish, the rice–fish farming system contains a wide variety of other organisms, including phytoplankton, zooplankton and microorganisms. The phytoplankton (>70%) mainly include Chlorophyta, Cyanophyta and Bacillariophyta; the zooplankton (>80%) mainly consist of Rotifera, Cladocera and Protozoa; the dominant phyla of microorganisms (>75%) are Proteobacteria, Actinobacteria and Bacteroidetes.

In the experiment, three stocking densities of largemouth bass were set: low stocking density (LD, 40 g/m^3^), medium stocking density (MD, 80 g/m^3^) and high stocking density (HD, 120 g/m^3^), and each density included three replicates. The average initial weight of the fish was 40.63 ± 0.13 g/fish. The fish were fed on a commercial pellet diet (Changzhou Haida Biological Feed Co., Ltd, Changzhou, China) which contained 47% crude protein, 5% crude lipids, 18.0% crude ash, 3.0% crude fiber, 10% water, 1.2% P and 2.7% lysine. The largemouth bass were fed two times per day, and the daily feed ration (approximately 2–3% of weight) was adjusted on their weight. During the experiment, DO and pH remained at 7.25 ± 0.32 °C and 5.8 ± 0.51 mg/L, respectively; total ammonia nitrogen and NO_2_-N never exceeded 0.5 mg/L and 0.1 mg/L, respectively. Any mortality of fish was recorded during the course of the experiment.

To evaluate the growth performance, the body weight and body length were measured on 30 August, 30 September and 30 October by randomly capturing 90 individuals per group. After 90 days of farming, 36 individuals (after 24 h of fasting) were randomly obtained from each group and anesthetized immediately using 50 mg/L of tricaine methane sulfonate (MS-222, Sigma, MO, USA), and then the blood and liver tissues were collected according to regular experimental operations. The tissues from 3 fish were mixed into one sample. The plasma was separated from the blood by centrifugation (3500 r/min, 10 min) and used to detect the physiological parameters and oxidative stress indices. The liver tissue was used to measure hepatic antioxidant status and the transcriptome sequence. The fish used in the study were permitted by the Freshwater Fish Research Center, and animal welfare was taken into consideration in all experimental operations.

### 2.2. Growth Performance

The growth performance was assessed by evaluating the following parameters: mean body weight, mean body length, condition factor = 100 × body weight/body length^3^, weight gain rate (WGR) = (average final weight—average initial weight)/average initial weight, specific growth rate (SGR) = 100 × (ln average final weight—ln average initial weight)/number of days, survival = 100 × final number of fish/initial number of fish, and feed conversion ratio (FCR) = food consumption/biomass increment.

### 2.3. Measurement of Physiological Parameters

Physiological parameters including glucose (Glu), total cholesterol (TC), triglyceride (TG), total protein (TP), albumin (Alb), lactate dehydrogenase (LDH), alanine aminotransferase (ALT), aspartate aminotransferase (AST), low-density lipoprotein cholesterol (LDL-c), high-density lipoprotein cholesterol (HDL-c) and alkaline phosphatase (AKP) in the plasma were measured using an automatic biochemical analyzer (BS-400, Shenzhen Mindray Bio-medical Electronics Co., LTD, Shenzhen, China). Lactate (LA) content in the plasma was detected by a colorimetric method using a commercial kit (Nanjing Jiancheng Bioengineering Institute, Nanjing, China). The levels of heat shock protein 70 (HSP 70), complement 3 (C3) and lysozyme (LZM) in the plasma were determined by an enzyme-linked immunosorbent assay (mlbio, Shanghai, China).

### 2.4. Measurement of Oxidative Stress Parameters

In plasma and liver tissues, the oxidative stress parameters including total antioxidant capacity (T-AOC), glutathione peroxidase (Gpx), superoxide dismutase (SOD), catalase (CAT), glutathione (GSH) and malondialdehyde (MDA) were measured using commercial kits. Kits for T-AOC, SOD, Gpx and MDA were purchased from Suzhou Grace Biotechnology Co., Ltd. (Suzhou, China), the CAT kit was provided by Beyotime Biotechnology (Shanghai, China) and the GSH kit was ordered from Nanjing Jiancheng Bioengineering Institute. T-AOC levels were measured by the ferric reducing ability of plasma (FRAP) method, where the antioxidants reduced Fe^3+^-tripyridine-triacridine (Fe^3+^-TPTZ) to produce blue Fe^2+^-TPTZ [35]. Gpx activity was detected using Cum-OOH as a substrate and 5,5′-dithiobis (2-nitrobenzoic acid) (DTNB) as a chromogenic agent [36]. SOD activity was analyzed by measuring the content of the reaction product (formazan) of water-soluble tetrazolium-8 (WST-8) and O^−^ [37]. CAT activity was tested by measuring the oxidation product of the decomposition of H_2_O_2_ under peroxidase [38]. GSH content was estimated by the DTNB method [39], MDA content was measured by the thiobarbituric acid (TBA) method [40] and hepatic protein content was tested by a bicinchoninic acid (BCA) assay [41].

### 2.5. Transcriptome Sequencing and Analysis

For the transcriptome study, we collected the livers of *M. salmoides* from the LD (named LL) and HD groups (named HL). Thirty-six individuals were randomly obtained from each group, and the liver tissues from 12 fish were mixed into one sample.

Total RNA of the liver tissue was separated using a Trizol reagent kit (Invitrogen, Carlsbad, CA, USA). Its quality was evaluated by an Agilent 2100 Bioanalyzer (Agilent Technologies, Palo Alto, CA, USA) and an agarose gel electrophoresis method. The RNA was enriched with Oligo(dT) beads and then used to synthesize cDNA with a commercial kit (NEB 7530, New England Biolabs, Ipswich, MA, USA). The cDNA library was constructed through size selection and PCR amplification, and the resulting cDNA library was sequenced using Illumina Novaseq6000 in Gene Denovo Biotechnology Co. (Guangzhou, China).

The raw data obtained from the sequencing machines were filtered by fastp (version 0.18.0) to obtain high-quality clean reads [42]. The clean reads were mapped to the *M. salmoides* reference genome (assembly ASM1485139v1) using HISAT2. 2.4 [43]. The transcription abundance was expressed as a FPKM (fragment per kilobase of transcript per million mapped reads) value calculated by RSEM software [44]. Relationship analysis of the samples was assessed by principal component analysis (PCA). Differentially expressed genes (DEGs) between LL and HL were analyzed by DESeq2 software [45] with a threshold (false discovery rate (FDR) < 0.05 and absolute fold change ≥ 2). The DEGs were subjected to enrichment analysis with the GO (Gene Ontology) and KEGG (Kyoto Encyclopedia of Genes and Genomes) databases. Significantly enriched GO terms and KEGG pathways were defined according to a threshold (FDR ≤ 0.05). In addition, we identified whether a set of genes in specific KEGG pathways displayed significant differences between the LL group and HL group through gene set enrichment analysis (GSEA) [46]. Raw data of the transcriptome sequences are available at the Sequence Read Archive (SRA) database (No. PRJNA838235).

### 2.6. Quantitative Real-Time PCR (qPCR) Analysis

Total RNA of liver tissues was extracted using RNAiso Plus reagent (Takara Biomedical Technology (Beijing) Co., Ltd, Beijing, China) and was then used to synthesize cDNA with a primeScript™ RT reagent kit (Takara, RR047A). The expression of the target gene was detected via qPCR amplification using a qPCR kit (Takara, RR820A). The relative mRNA level was calculated by the 2^−^^△△^^Cq^ method [47] using β-actin as a reference gene. The specific primers of the target genes in the study are listed in Table S1.

### 2.7. Statistical Analysis

All data were analyzed using SPSS version 20.0 software and expressed as means ± SEM (standard error of mean). The Shapiro–Wilk test and Levene test were used to analyze the normal distribution and variance homogeneity of these data, respectively. The difference among different stocking densities was analyzed by one-way analysis of variance (ANOVA) with LSD (least significant difference). Differences between the LL group and the HL group were assessed by Student’s t-test. A *p*-value of <0.05 was considered statistically significant.

## 3. Results

### 3.1. Changes in Growth Performance

**Figure 1:**
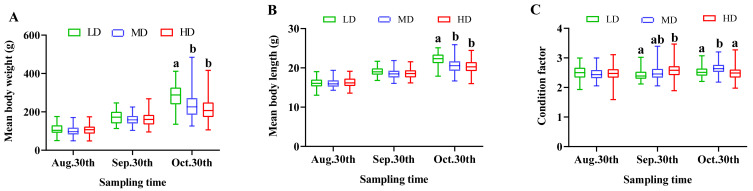
Mean body weight (**A**), mean body length (**B**) and condition factor (**C**) of *M. salmoides* reared at different densities in an integrated rice–fish farming system. Different letters as superscripts indicate significant differences among the different groups (*p* < 0.05). LD, low stocking density; MD, medium stocking density; HD, high stocking density.

In this experiment, the SR was 86.38 ± 1.82%, 79.02 ± 1.08% and 76.67 ± 1.05% in the LD, MD and HD groups, respectively (Table 1). The mean body weight and mean body length of *M. salmoides* showed a rising tendency during the course of the study, and the two parameters were markedly higher in the LD group than in the MD and HD groups on 30 October (*p* < 0.05; Figure 1A,B). The condition factor value in the HD group was higher than that in LD on 30 September, while it was higher in the MD group than in the other groups on 30 October (*p* < 0.05; Figure 1C). At the end of the experiment, the FBW, WGR and SGR were higher in the LD group than in the MD and HD groups (*p* < 0.05; Table 1), but the FCR was not changed by the different stocking densities (*p* > 0.05).

### 3.2. Changes in Physiological Parameters

The physiological parameters of *M. salmoides* in the different groups are shown in Table 2. The levels of ALT, AST, LDH, AKP, TP, HDL-c, Alb, HSP70 and LZM were similar in the LD, MD and HD groups after 90 days of farming (*p* > 0.05). The Glu and LA concentrations were significantly increased in the HD group compared with the LD group (*p* < 0.05). However, the TG, TC, LDL-c and C3 levels were significantly decreased in the HD groups compared with the LD group (*p* < 0.05). In addition, compared with the LD group, the LA value was increased but the LDL-c value was lower in the MD group (*p* < 0.05).

### 3.3. Changes in Oxidative Stress Parameters

**Figure 2:**
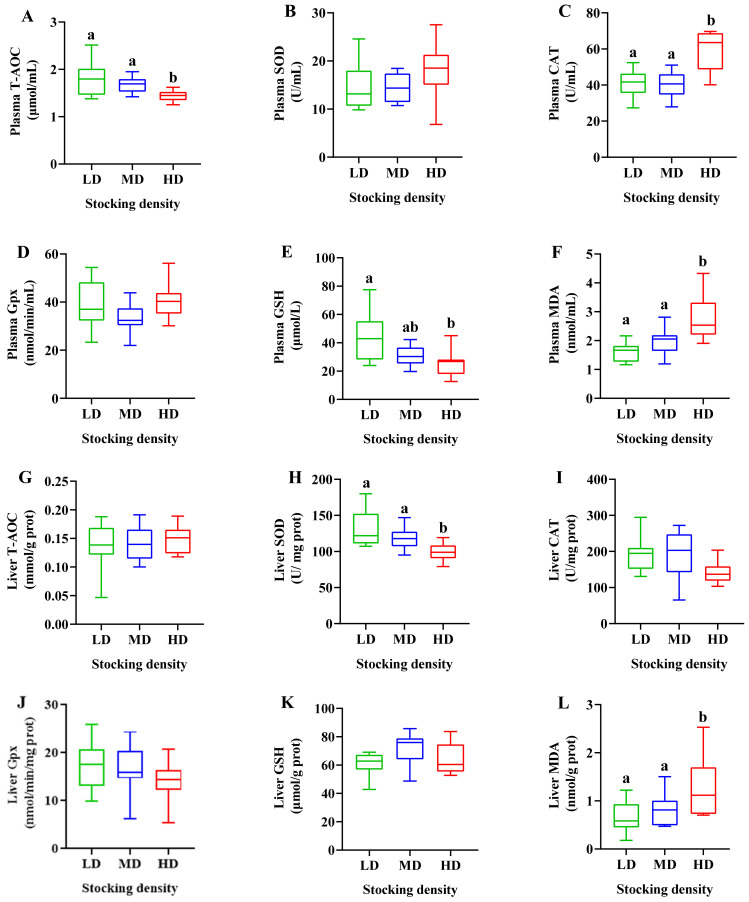
Antioxidative parameters in the plasma (**A**–**F**) and liver (**G**–**L**) of *M. salmoides* reared at different densities in an integrated rice–fish farming system after 90 days of farming. Values are expressed as means ± SEM, n = 12. Different letters as superscripts indicate a statistical significance among different densities (*p* < 0.05). LD, low stocking density; MD, medium stocking density; HD, high stocking density.

After 90 days of farming, the plasma T-AOC and GSH levels were significantly reduced in the HD group compared with the LD group, while the CAT and MDA levels were prominently enhanced in the HD group (*p* < 0.05; Figure 2A,C,E,F). SOD and Gpx activities in the plasma were not visibly influenced under different stocking densities (*p* > 0.05; Figure 2B,D). In the liver, the HD treatment clearly inhibited SOD activity and enhanced MDA formation compared with the LD and MD groups (*p* < 0.05; Figure 2H,L). In addition, the other oxidative stress parameters did not differ significantly in the liver among the three groups (*p* > 0.05; Figure 2G,I,J,K).

### 3.4. Transcriptome Sequencing and Analysis of DEGs

**Figure 3:**
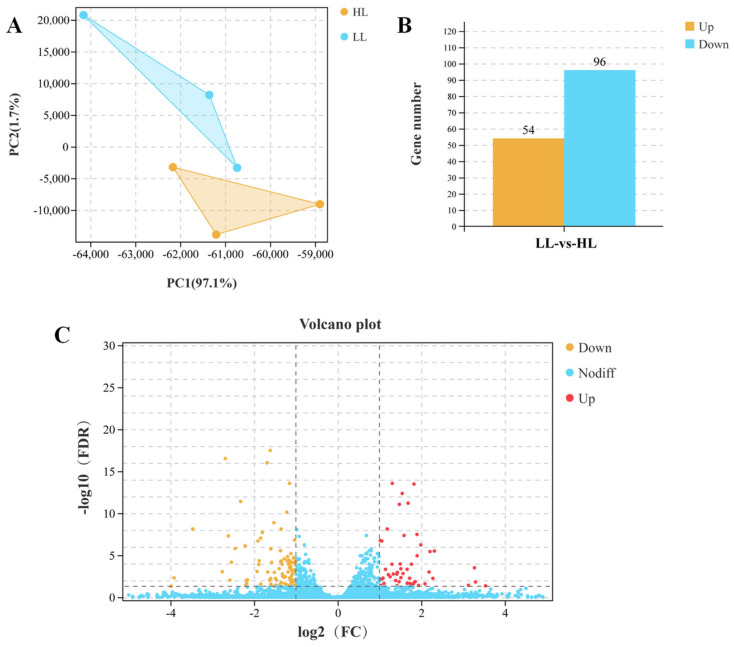
Differently expressed genes (DEGs) in the liver of *M. salmoides* between the low-density group (LL) and the high-density group (HL). (**A**) The correlation of samples between the LL group and the HL group. (**B**) The number of DEGs in the liver between the LL and HL groups. (**C**) Volcano plot of DEGs between the LL group and the HL group.

After filtering of raw reads, 40,491,348 (99.36%)—49,617,030 (98.94%) clean reads were obtained in the livers of the LL group and the HL group. The base quality score indicated that the values of Q30, Q20 and GC were 90.11–94.07%, 96.09–97.91% and 48.08–49.10%, respectively. The mapped ratio was greater than 93.36%, and 25,050 genes were identified according to alignment against the reference sequence (Table S2). According to the expression level of each gene in the samples, the PCA revealed distinct groups between the LL group and HL group (Figure 3A). After 90 days of farming, there were 150 DEGs, including 54 upregulated genes and 96 downregulated genes (Figure 3B,C).

### 3.5. GO Enrichment Analysis of DEGs

**Figure 4:**
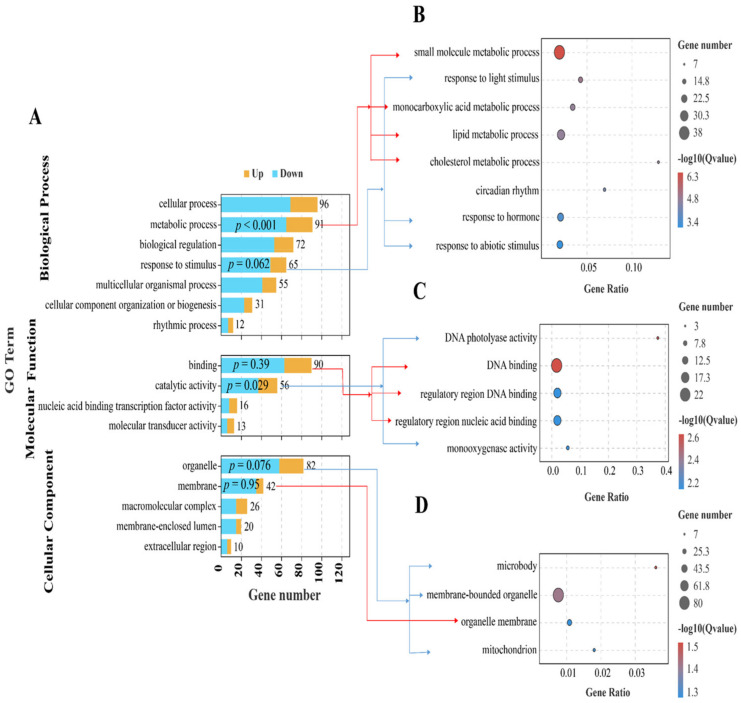
GO enrichment analysis for differently expressed genes (DEGs) in the livers of *M. salmoides* between the low-density group (LL) and the high-density group (HL). (**A**) GO enrichment terms pf DEGs in biological processes, molecular functions and cellular components. (**B**) Top 8 GO terms in the biological process category. (**C**) Top 5 GO terms in the molecular function category. (**D**) Top 4 GO terms in the cellular component category. *P*-values were corrected via the Benjamin–Hochberg method.

For an evaluation of the biological function, we performed GO enrichment analysis on the DEGs (Figure 4). In the biological process category, the DEGs were enriched mainly in cellular processes, metabolic processes (*p*.adjust < 0.001), biological regulation and response to stimulus, and specifically, in single-organism metabolic processes (*p*.adjust < 0.001), lipid metabolic processes (*p*.adjust < 0.001) and the response to light stimulus (*p*.adjust < 0.001) (Figure 4B). In the molecular function category, two major Level 2 GO terms were binding and catalytic activity (*p*.adjust = 0.029). In detail, the DEGs were primarily involved in DNA photolyase activity (*p*.adjust = 0.002), DNA binding (*p*.adjust = 0.002) and monooxygenase activity (*p*.adjust = 0.007) (Figure 4C). In the cellular components category, organelles and membranes contained the most DEGs, and microbodies (*p*.adjust = 0.028) and membrane-bounded organelles (*p*.adjust = 0.036) were the top two GO terms (Figure 4D).

### 3.6. KEGG Enrichment Analysis of DEGs

**Figure 5:**
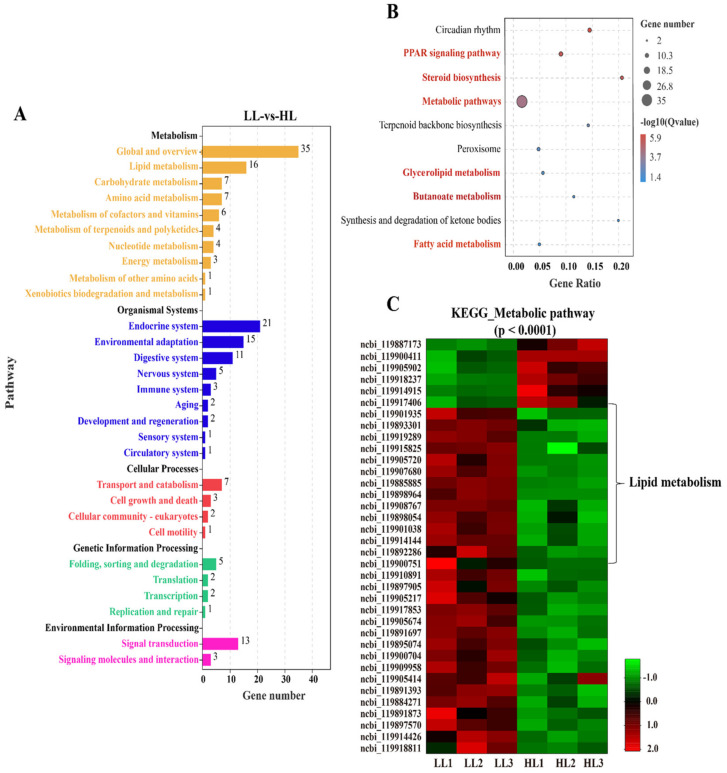
KEGG enrichment analysis for differently expressed genes (DEGs) in the liver of *M. salmoides* between the low-density group (LL) and the high-density group (HL). (**A**) DEG enrichment in the KEGG A Class and B Class. (**B**) The top 10 enriched KEGG pathways. (**C**) DEGs enriched in the metabolic pathway.

To explore the key signaling pathways, the DEGs were further annotated against the KEGG database (Figure 5). The DEGs were enriched in five KEGG A classes: metabolism, organismal systems, cellular processes, genetic information processing and environmental information processing (Figure 5A). The top 10 KEGG pathways indicated that the DEGs were mainly enriched in lipid metabolism-related pathways, including the PPAR signaling pathway, steroid biosynthesis, glycerolipid metabolism and fatty acid metabolism (Figure 5B). In addition, we observed that 36 genes were enriched in the metabolic pathway (q value < 0.0001), of which 16 genes were involved in lipid metabolism (Figure 5C).

### 3.7. Changes in the PPAR Signaling Pathway and Lipid Metabolism

**Figure 6:**
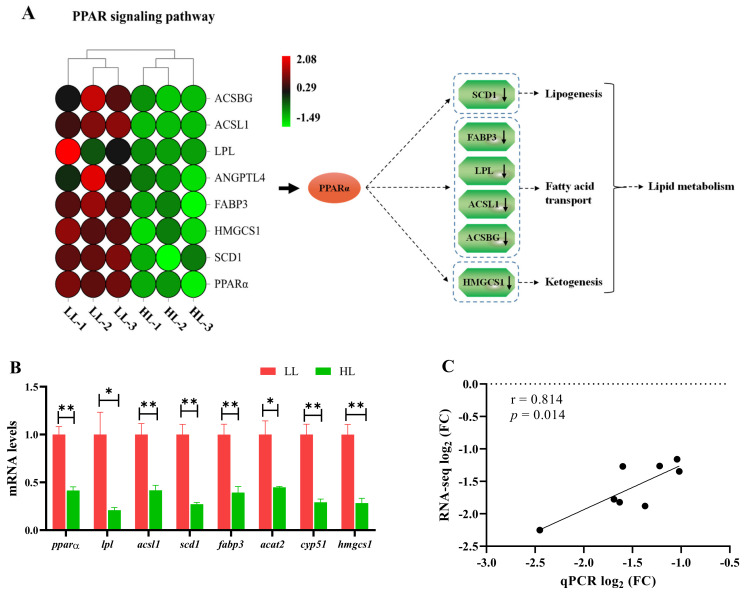
Changes in the PPAR signaling pathway and lipid metabolism-related genes in the liver of *M. salmoides* between the low-density group (LL) and the high-density group (HL). (**A**) DEGs in the KEGG PPAR signaling pathway and possible mechanisms regulating lipid metabolism. (**B**) Lipid metabolism-related gene expression measured by qPCR; values are expressed as means ± SEM (n = 3), * *p* < 0.05 and ** *p* < 0.01. (**C**) The correlation between the qPCR data and RNA-seq data.

At the end of the experiment, the PPAR signaling pathway in the liver was considerably altered (*p* < 0.0001; Figure 6A). All altered genes showed a downregulated tendency in the HL group compared with the LL group, including the key genes *pparα*, *scd1*, *fabp3*, *lpl*, *acsl1* and *hmgcs1* (Figure 6A,B). Further, the key genes involved in the PPARα signaling pathway and lipid metabolism were verified by qPCR (Figure 6B), and the results showed that the RNA-seq data were significantly consistent with the qPCR data (*r* = 0.814, *p* = 0.014; Figure 6C).

### 3.8. Changes in Lipid Metabolism-Related Pathways

**Figure 7:**
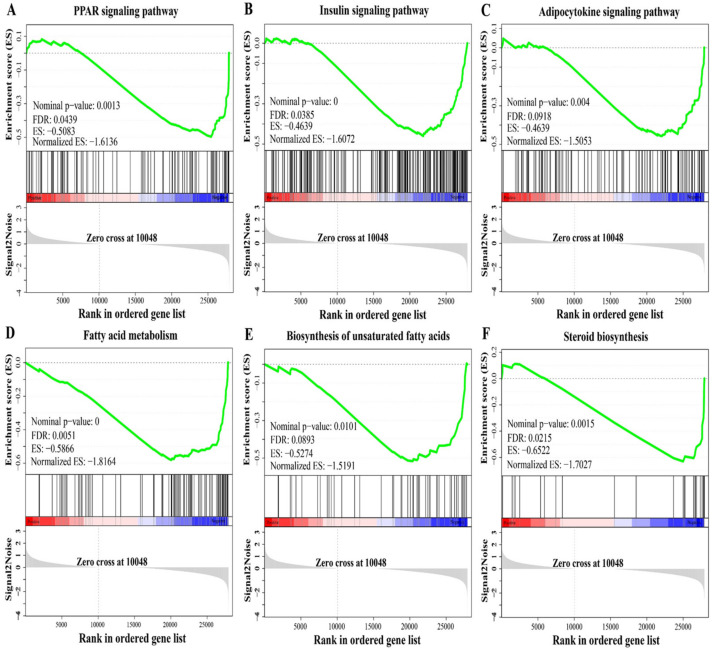
Changes in lipid metabolism-related pathways identified using GSEA in the liver of *M. salmoides* between the low-density group (LL) and the high-density group (HL). (**A**) PPAR signaling pathway. (**B**) Insulin signaling pathway. (**C**) Adipocytokine signaling pathway. (**D**) Fatty acid metabolism. (**E**) Biosynthesis of unsaturated fatty acids. (**F**) Steroid biosynthesis. The |Normalized ES| > 1, nominal *p*-value < 0.05 and FDR < 0.25 in each gene set were set as threshold values for statistical significance.

The GSEA showed significant enrichment in a range of pathways related to lipid metabolism including the PPAR signaling pathway (KO03320), the insulin signaling pathway (KO04910), the adipocytokine signaling pathway (KO04920), fatty acid metabolism (KO01212), steroid biosynthesis (KO00100) and biosynthesis of unsaturated fatty acids (KO01040) (Figure 7). The coordinated expression changes in the six pathways were significantly negatively regulated by the HL treatment. These results were consistent with the results of KEGG enrichment of the DEGs.

## 4. Discussion

The stocking density is a critical factor affecting the health status and production of farmed fish. Increasing the stocking density is a positive means of improving fish production and water utilization. However, an excessive stocking density may result in adverse effects for farmed fish, such as inhibiting growth performance, deteriorating water quality, and increasing mortality and disease risk. Therefore, an appropriate stocking density is required for farmed fish under different farming models. In the present study, we set out to evaluate the growth and physiological effects of different stocking densities in *M. salmoides* under an integrated rice–fish farming model, followed by assessing the underlying mechanisms via RNA-seq analysis. Accordingly, our experiment indicated that the high stocking density caused adverse changes in the growth performance, physiological responses, antioxidant status and hepatic metabolic function of *M. salmoides*.

### 4.1. Effect of Stocking Density on Growth Performance

Reduced growth performance is a common phenomenon for farmed fish under high stocking densities. Numerous studies have reported that the growth performance was negatively influenced in different fish species when the stocking density was above a certain value. In *Lates calcarifer*, the body weight and feed utilization efficiency were significantly decreased with an increasing stocking density (350−1750 g/m^3^) after 60 days of farming [48]. In *Piaractus mesopotamicus*, the low stocking density (650 g/m^3^) group had a higher final biomass, weight gain and economic profit than the high stocking density group (1300 g/m^3^) after 360 days of faming [49]. Similarly, in this work, the HD and MD treatments reduced the body length, body weight, WGR and SGR of *M. salmoides* after 90 days of farming, which indicated that a density of up to 375 g/m^3^ (the final density of the MD group) had an adverse impact on growth performance in the integrated rice–fish system. It is worth noting that the tolerance of *M. salmoides* to high stocking density shows obvious differences in different farming models. In a land-based recirculating aquaculture system, the growth performance of *M. salmoides* decreased when the density reached up to 23.34 kg/m^3^ (65 times as high as our data) [28]. In a pond system, *M. salmoides* reared at a high stocking density (about 798 g/m^2^) showed lower growth [50]. The low growth performance seemed to be related to inappropriate environmental and/or feeding conditions, the reduction in available space for individuals and the extra energy requirements induced by stress under a high stocking density [51,52,53].

The survival ratio is a crucial index for evaluating production and the economic benefit of farmed fish. Previous studies have shown that higher densities can cause higher mortality rates in some cultured species, such as *Platax teira* and *O. niloticus* [54,55]. In an in-pond raceway culture system, the survival of *M. salmoides* showed a downward tendency with an increasing density [56]. In the present work, we also found that the survival ratio of *M. salmoides* was significantly higher in the LD group than in the MD and HD groups. In addition, the average survival ratio (80.87%) of *M. salmoides* in the rice–fish farming system was slightly higher than that (74.47%) in pond-based production systems [57].

### 4.2. Effect of Stocking Density on Physiological Function

The physiological response is closely associated with the growth performance of farmed fish in aquaculture, which is commonly used to assess fish heath and to optimize management measures. High stocking density, as a chronic stressor, has been known to directly influence the physiological response of some teleost species [58,59]. Clu and LA were considered as reliable indicators for evaluating the stress response of fish. Under stress conditions, the blood Clu and LA levels were significantly increased, which agree with high energy consumption [60]. The increase in blood Glu and LA in fish is a common response to stress under high stocking densities [17]. In this study, higher plasma Glu and LA concentrations were also observed in the HD group, suggesting a physiological response to stress induced by the high stocking density. These results were supported by previous studies in *Sparus aurata*, *Solea senegalensis* and *Lates calcarife* [16,61,62]. In fish, HSP70 was also identified as a sensitivity indicator of stress, mediating protein folding and repairing. The high expression of the *hsp* 70 gene has been reported in fish farmed at a high density, as it is protective against the adverse environment [61,62]. However, in the rice–fish system, there was no significant difference in plasma HSP70 content among different stocking densities, suggesting that the current density may not have achieved stress levels that led to a change in HSP70.

In addition to Glu and LA, the TC and TG in the blood are also common parameters for the assessment of fish stress, as these are related to energy and lipid metabolism. It has been confirmed that high stocking densities affect energy reserves, consumption and reallocation to cope with the increased energy demand in fish [53]. In *Megalobrama amblycephala*, the TG and TC, as energy substrates, were markedly increased at a high stocking density [58]. Similarly, the high stocking density treatment increased the TC and TG in *Ictalurus punctatus*, which was linked to augmented energy metabolism [63]. However, in our study, the TC and TG levels displayed a decreasing trend with increasing density. We speculated that decreased the TC and TG were linked to an abnormal lipid metabolism in the liver under HD conditions. Consistent with our data, lower levels of TG and TC were also reported in *O. mykiss* and *Trachinotus ovatus* cultured at a higher stocking density, and the authors suggested that lipid reserves were depleted in response to the increased energy demand under the stress conditions [61,64].

Abnormal immune function has been frequently reported in fish farmed at a high density. LZM and C3 are commonly used to evaluate immune status in fish after adverse stimuli. Costas et al. (2013) suggested that the decrease in plasma LZM and ACH50 displayed an impairment of the immune system in *Solea senegalensis* held at a high stocking density [65]. Liu et al. (2019) postulated that the decreased C3 and LZM indicated immune suppression in *Scophthalmus maximus* under a high stocking density [66]. Similarly, in the present work, the C3 level was decreased in the HD group after 90 days of farming, revealing that the high density had an adverse effect on the immunity of *M. salmoides*. In addition, our data showed that the GOT, GPT, AKP and LDH levels were not changed by the high stocking density, suggesting that the current density did not cause tissue damage [67].

### 4.3. Effect of Stocking Density on Antioxidative Status

An increasing amount of evidence has suggested that a high stocking density can induce intracellular redox imbalance, leading to oxidative stress or oxidative damage in many fish species [68,69]. In order to defend against oxidative stress, fish, similar to mammals, have developed an antioxidant defense system consisting of enzymatic antioxidants (e.g., SOD, Gpx and CAT) and non-enzymatic antioxidants (e.g., GSH, Vitamin C, carotenoids and flavonoids) [70]. It can be activated to maintain redox status under moderate oxidative stress [71]. Nevertheless, the antioxidant defense system is impaired and antioxidants are depleted under chronic or severe oxidative stress [72]. Indeed, several previous studies have shown that a high stocking density treatment decreased the levels of antioxidant parameters, such as SOD, GSH and CAT, leading to impairment of the antioxidant defense system in *O. mykiss*, *O. niloticus* and *S. maximu* [13,19,20]. Moreover, a study on *M. salmoides* farmed in an in-pond raceway system showed that high stocking density reduced hepatic SOD and CAT activity, and restrained antioxidant capacity [27]. Conversely, increased levels of the antioxidants were also observed in fish under high density, suggesting a positive response of the antioxidant defense system to stress [73,74]. Interestingly, in this study, the levels of plasma T-AOC and GSH were suppressed, but the plasma CAT activity was enhanced in HD group, suggesting a depletion of non-enzymatic antioxidants and an adaptive response to adverse conditions in plasma. Moreover, in the liver, only SOD activity was inhibited in the HD group, but the other antioxidant parameters were not significantly changed, which may indicate moderate or slight oxidative stress in the livers of *M. salmoides* reared in the HD group.

Lipid peroxidation is a vital consequence of oxidative stress, usually reflected by MDA content. Previous studies have confirmed that high stocking density as a stressor can stimulate the formation of ROS in fish [73]. Excess ROS may react with unsaturated fatty acids on cell membranes, inducing lipid peroxidation [75]. It has been reported that a large number of harmful byproducts from lipid peroxidation are capable of inactivating many cellular proteins, inducing inflammation and damaging cells or tissues [76]. In fish, increased lipid peroxidation has always attracted widespread attention under high stocking densities. We and others have found that the high stocking density treatment promoted MDA formation in the liver, blood or intestines of fish [13,19,20,77]. Similarly, increased MDA content was also observed in the plasma and liver of *M. salmoides*, indicating that the high-density treatment induced lipid peroxidation after 90 days of farming in the rice–fish farming system.

### 4.4. Effect of Stocking Density on Lipid Metabolism

Liver is a major lipid metabolism organ of fish, and lipid metabolism is more susceptible to environmental stressors, as it is a major energy substrate [77]. The adverse effect of a high stocking density on lipid metabolism has been widely reported in fish. Earlier studies have found that high stocking density caused a decrease in hepatic lipid content, suggesting a higher utilization of hepatic lipids under stressful conditions [78,79]. Under a high stocking density, lipid metabolic enzymes, including fatty acid synthetase, hormone-sensitive triglyceride lipase and lipoprotein lipase, were upregulated in the liver of *O. niloticus* to adapt to adverse conditions [80]. Transcriptomic analysis showed that *Ctenopharyngodon idella* farmed at a high stocking density might face issues associated with abnormal lipid metabolism [81]. Metabolomic analysis showed that lipid metabolism was repressed by a high stocking density in *Brachymystax lenok* [82]. In the current study, our data also revealed that the lipid metabolic process in the GO enrichment analysis and the lipid metabolism-related pathways in the KEGG enrichment analysis were significantly altered in the HD group, where the fatty acid metabolism, biosynthesis of unsaturated fatty acids and steroid biosynthesis tended to be suppressed in the liver of *M. salmoides*. The suppression may be harmful for the growth of *M. salmoides*, since fatty acids are essential for multiple physiological functions, such as energy production and membrane structure [83].

It was worth noting that we also found three key pathways related to lipid metabolism, including the PPAR signaling pathway, the insulin signaling pathway and the adipocytokine signaling pathway, which showed a significant difference between the LL group and the HL group. PPARα is regarded as a master regulator in lipid metabolism, which can activate its target genes (e.g., *scd*, *fabp*, and *acsl*) to mediate fatty acid transport, synthesis and oxidation, as well as lipogenesis and ketogenesis [84]. Its activation improves the symptoms of metabolic syndrome and exerts anti-inflammatory activity [85]. Ren et al. (2017) suggested that upregulation of PPARα under a high stocking density enhanced lipid mobilization and utilization in *Acipenser schrenckii* to cope with crowding stress [86], but Xu et al. (2020) found that PPARα expression was downregulated in *Takifugu rubripes* under a high density, revealing a decrease in fatty acid β-oxidation and lipid metabolism disorder [87]. In this study, downregulation of PPARα further inhibited its downstream genes, including *sce1*, *lpl*, *fabp3*, *acsl1*, *hmgcs1* and *angptl4*, indicating that the abnormal lipid metabolism in the HD group was associated with the suppression of the PPARα signaling pathway.

Growing evidence has demonstrated that the insulin signaling pathway is involved in lipid metabolism. Its activation can stimulate lipid biosynthesis [88] and regulate lipid deposition by activating lipogenesis and inhibiting fatty acid oxidation [89]. Multi-omics analyses revealed that the insulin signaling pathway was downregulated in the liver of *O. niloticus* under hypoxia stress [90]. In our work, the GSEA data were more inclined to indicate a downregulated insulin signaling pathway in the liver of *M. salmoides* under the HD treatment. In addition, the insulin signaling pathway is essential for normal glucose metabolism, and its upregulation could promote glucose metabolism [91,92,93]. Therefore, the downregulated insulin signaling pathway may adversely influence glucose metabolism and energy utilization in the liver.

The adipocytokine signaling pathway is a signaling cascade arising from the adipocytokines, which has been implicated in energy intake, fatty acid metabolism and insulin resistance [94]. Multi-omics analyses identified that the adipocytokine signaling pathway was related to fatty acid metabolism in *Cyprinus carpio* [95]. Microarray analysis found that the adipocytokine signaling pathway was significantly changed in *Danio rerio* under heat stress [96]. The activated adipocytokine signaling pathway might be further involved in the immune response in *Ctenopharyngodon idella* after lipopolysaccharide exposure [97]. In this study, the adipocytokine signaling pathway was negatively affected by the HD treatment, which might be a cause of hepatic lipid metabolism disorder.

### 4.5. Effect of Stocking Density on Welfare

Welfare is an increasing public concern in farmed fish. Accumulating evidence suggests that fish experience poor welfare under adverse conditions, accompanied by altered growth performance, physiological parameters and behavior [98]. The stress response is a common event when fish are subjected to poor welfare, and thus, physiological indicators of stress, including cortisol, glucose and lactate, are frequently used to evaluate the welfare of farmed fish [99]. A recent study also suggested that HSP 70 was a new candidate for assessing the welfare of *M. salmoides* [100]. In addition, there is a significant negative correlation between poor welfare and growth performance; therefore, the growth rate is also considered as a reliable indicator in monitoring stress and welfare [101]. Under stress conditions, energy metabolism increases to cope with abnormal physiological changes, which lead to a decrease in energy availability for growth [102]. Stocking density has been highlighted as a principal area of welfare concern in farmed fish, because they are often held at higher densities in commercial aquaculture [103]. An earlier study reported that high stocking density had the potential to adversely affect the welfare of *O. mykiss*, which was assessed by measuring population, individual, morphometric and physiological indicators [104]. A multivariate analysis showed that increasing stocking densities were associated with lower welfare score in *Salmo salar* [105]. In *Clarias gariepinus*, a high stocking density impaired welfare via decreasing growth performance and increasing skin lesions [17,106]. In line with previous studies, we also monitored the welfare through measuring the growth performance and physiological indicators (Glu, LA and HSP 70), and the data showed a poor welfare in *M. salmoides* in the HD group, reflected by a low growth rate, higher Glu and LA levels, and abnormal lipid metabolism.

## 5. Conclusions

In conclusion, our data indicated that the *M. salmoides* can be reared in integrated rice–fish farming systems. However, when the stocking density reached 375 g/m^3^ (the final density in the MD group at the current size), the growth performance was negatively influenced. The high stocking density, as a chronic stressor, induced a physiological response and oxidative stress, which may have resulted in greater energy consumption and growth inhibition. Further, the transcriptome analysis showed that the HD treatment caused abnormal hepatic lipid metabolism via suppressing the PPAR, insulin and adipocytokine signaling pathways, which may be another potential cause of the low growth in the HD group. Thus, in a commercial integrated rice–fish farming system, improvements in strategies for managing *M. salmoides* regarding stocking density are required. This study provides a valuable reference for *M. salmoides* farming and optimization of the rice–fish farming model.

## Figures and Tables

**Table 1:** Growth parameters of *M. salmoides* reared at different densities in an integrated rice–fish farming system after 90 days of farming.

Parameters	LD	MD	HD
IBW (g)	40.76 ± 0.25	40.67 ± 0.21	40.43 ± 0.19
FBW (g)	278.82 ± 7.62 ^a^	236.68 ± 7.68 ^b^	213.81 ± 5.29 ^b^
WGR (%)	582.67 ± 14.38 ^a^	483.16 ± 18.07 ^b^	429.47±12.63 ^b^
SGR (%/d)	2.18 ± 0.024 ^a^	2.01 ± 0.035 ^b^	1.89 ± 0.056 ^b^
SR (%)	86.38 ± 1.82 ^a^	79.02 ± 1.08 ^b^	76.67 ± 1.05 ^b^
FCR	1.13 ± 0.019	1.12 ± 0.035	1.22 ± 0.049

Values are expressed as mean ± SEM of three replicates. For each row, means with different letters as superscripts indicate a statistical significance among different densities (*p* < 0.05). LD, low stocking density; MD, medium stocking density; HD, high stocking density.

**Table 2:** Physiological parameters in the plasma of *M. salmoides* reared at different densities in an integrated rice–fish farming system after 90 days of farming.

Parameters	LD	MD	HD
ALT (U/L)	4.48 ± 0.52	3.30 ± 0.39	2.84 ± 0.36
AST (U/L)	18.62 ± 2.00	17.36 ± 1.80	19.55 ± 2.37
LDH (U/L)	278.97 ± 32.07	295.08 ± 37.84	235.31 ± 23.14
AKP (U/L)	33.19 ± 1.21	33.44 ± 1.36	31.70 ± 2.35
TP (g/L)	39.49 ± 0.39	37.23 ±1.26	35.08 ± 1.65
Alb (g/L)	8.38 ± 0.60	8.86 ± 0.46	8.56 ± 0.40
Glu (mmol/L)	5.26 ± 0.20 ^a^	5.64 ± 0.38 ^a^	8.98 ± 0.46 ^b^
LA (mmol/L)	7.51 ± 0.20 ^a^	8.72 ± 0.21 ^b^	9.18 ± 0.20 ^b^
HSP70 (pg/mL)	49.79 ± 5.05	45.98 ± 5.02	39.06 ± 2.64
TC (mmol/L)	12.00 ± 0.26 ^a^	11.07 ± 0.45 ^ab^	9.40 ± 0.48 ^b^
TG (mmol/L)	18.46 ± 0.94 ^a^	17.71 ±0.47 ^a^	13.36 ± 0.55 ^b^
LDL-c (mmol/L)	3.78 ±0.12 ^a^	2.94 ± 0.13 ^b^	2.39 ± 0.14 ^b^
HDL-c (mmol/L)	3.81 ± 0.09	3.65 ±0.17	3.57 ± 0.20
C3 (μg/mL)	9.32 ± 0.70 ^a^	10.74 ± 1.22 ^a^	5.75 ± 0.61 ^b^
LZM (ng/mL)	32.71 ± 3.44	36.77 ± 5.63	26.77 ± 2.78

Values are expressed as means ± SEM, n = 12. For each row, means with different letters as superscripts indicate a statistical significance among different densities (*p* < 0.05). LD, low stocking density; MD, medium stocking density; HD, high stocking density.

## Data Availability

The raw data of transcriptome sequencing are available at the Sequence Read Archive (SRA) database of NCBI (No. PRJNA838235).

## Supplementary Materials

The following supporting information can be downloaded at: https://www.mdpi.com/article/10.3390/antiox11071215/s1. Figure S1: Schematic diagram of the integrated rice–fish farming system in this study. Table S1: Specific primer sequences for qPCR in this study. Table S2: Valid data used in the transcriptome analysis.
