# Supplementary material for: Effects of Stocking Density on the Growth Performance, Physiological Parameters, Redox Status and Lipid Metabolism of Micropterus salmoides in Integrated Rice–Fish Farming Systems
Source: Antioxidants (Basel). 2022 Jun 21;11(7):1215. doi: 10.3390/antiox11071215 (PMC9312047; doi:10.3390/antiox11071215)
Supplement: Supplementary file 1 [file antioxidants-11-01215-s001.zip › antioxidants-1754031-supplementary.pdf]

Supporting Information

Table S1. Specific primer sequences for qPCR in the study

Gene	Primer sequence(5'-3')
peroxisome proliferator-activated receptor alpha (pparα)	F: TGCCAGAGTACCTCAGTGGA
	R: GGCCGTCTTTGTTCATGCAG
lipoprotein lipase (*lpl*)	F: CCTGCTAGTGACGGAGACG
	R: CCATCTTTTGTTGGGTCTCGC
long-chain acyl-CoA synthetase (*acsl*)	F: ACTGGTGGATGTGCCAGAAA
	R: TCAGAGTGCCGTTAGGAAGC
stearoyl-CoA desaturase 1 (*scd1*)	F: TGCTGAGGTACACGTTGGTG
	R: TCTTGTCGTAGGGCCTGTTTC
fatty acid-binding protein 3 (*fabp3*)	F: AGAGCACCTTCAAAACTACCGA
	R: TTCTTTGCCGTCCCAGCTTT
sterol 14alpha-demethylase (*cyp51*)	F: TGCCACCTACAAAGACGGAC
	R: CAGGTCTTCTCCACAGACGG
acetyl-CoA C-acetyltransferase (acat2)	F: AGCAGCAGCAACTGTCCTAAT
	R: CCTGCTTTCTCAACCGCTTTC
hydroxymethylglutaryl-CoA synthase (hmgcs1)	F: TACGGATCAGGATTTGCTGC
	R: ATTGGCTAGGTGATGCGTCT
β-actin	F: AAAGGGAAATCGTGCGTGAC
	R: AAGGAAGGCTGGAAGAGGG

Table S2 Valid data used in transcriptome analysis

Samples	Raw reads	Clean Reads (%)	Q20 (%)	Q30 (%)	GC (%)	Mapping ratio (%)	Sequenced gene
LL-1	50,146,234	98.94	96.09	90.25	48.08	93.36	18,512
LL -2	49,525,544	99.15	96.23	90.52	48.53	93.91	19,956
LL -3	41,643,142	99.57	97.88	93.98	48.33	96.83	19,718
HL-1	48,339,076	99.13	96.43	90.95	48.63	93.92	19,502
HL-2	40,753,914	99.36	96.07	90.11	49.10	93.51	19,330
HL-3	45,075,010	99.34	97.91	94.07	48.88	97.14	19,815

GC, GC content in clean reads, Q20 and Q30 the base quality score (Q score) was no less than 20 and 30, respectively, in clean reads. LL, Low density group; HL, High density group.

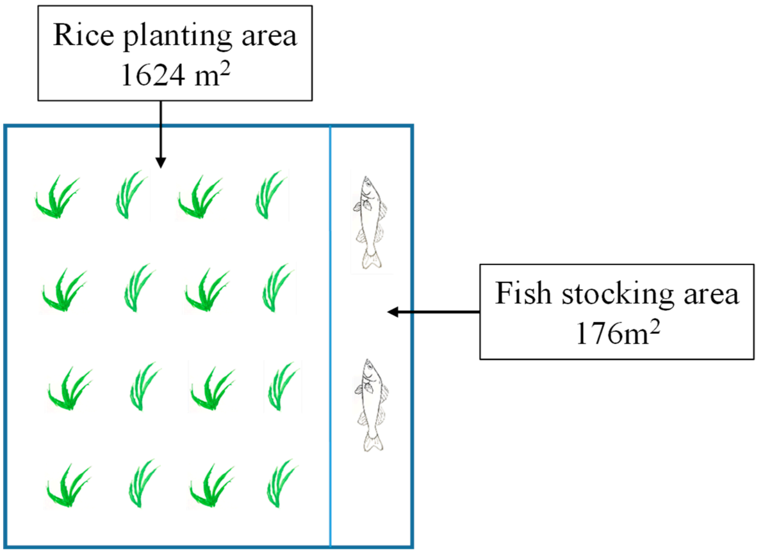

Figure S1. Schematic diagram of integrated rice-fish farming system in this study
